# Abundant Fas expression by gastrointestinal stromal tumours may serve as a therapeutic target for MegaFasL

**DOI:** 10.1038/sj.bjc.6604736

**Published:** 2008-10-21

**Authors:** B Rikhof, W T A van der Graaf, C Meijer, P T K Le, G J Meersma, S de Jong, J A Fletcher, A J H Suurmeijer

**Affiliations:** 1 Department of Medical Oncology, University Medical Center Groningen, University of Groningen, Hanzeplein 1, Groningen 9713 GZ, The Netherlands; 2 Department of Medical Oncology, Radboud University Nijmegen Medical Centre, Geert Grooteplein-Zuid 10, Nijmegen 6525 GA, The Netherlands; 3 Department of Pathology, Brigham and Women's Hospital, 75 Francis Street, Boston, MA 02155, USA; 4 Department of Pathology, University Medical Center Groningen, University of Groningen, Hanzeplein 1, Groningen 9713 GZ, The Netherlands

**Keywords:** gastrointestinal stromal tumour, Fas, Fas ligand, imatinib, MegaFasL

## Abstract

Although the tyrosine kinase inhibitor imatinib has been shown to be an active agent in patients with gastrointestinal stromal tumours (GIST), complete remissions are almost never seen and most patients finally experience disease progression during their course of treatment. An alternative therapeutic option is to target death receptors such as Fas. We showed that a panel of imatinib-sensitive (GIST882) and imatinib-resistant (GIST48, GIST430 and GIST430K-) cell lines expressed Fas. MegaFasL, a recently developed hexameric form of soluble Fas ligand (FasL), appeared to be an active apoptosis-inducing agent in these cell lines. Moreover, MegaFasL potentiated the apoptotic effects of imatinib. Immunohistochemical evaluations, in 45 primary GISTs, underscored the relevance of the Fas pathway: Fas was expressed in all GISTs and was expressed strongly in 93%, whereas FasL was expressed at moderate and strong levels in 35 and 53% of GISTs, respectively. Fas and FasL expression were positively correlated in these primary GISTs, but there was no association between Fas or FasL expression and primary site, histological subtype, tumour size, mitotic index, risk classification, and *KIT* mutation status. The abundant immunohistochemical Fas and FasL expression were corroborated by western blot analysis. In conclusion, our data implicate Fas as a potential therapeutic target in GIST.

The observation that nearly all spindle cell and epithelial tumours of the stomach and bowel highly express the receptor tyrosine kinase KIT has led to the characterisation of gastrointestinal stromal tumours (GISTs) as a distinct clinicopathological entity different from other gastrointestinal mesenchymal tumours. Various *KIT* genomic mutations occur in about 80% of GISTs. In addition, about 5% of GISTs have mutations in the platelet-derived growth factor receptor-*α* (PDGFRA) (Corless *et al*, 2004). These mutations lead to ligand-independent activation of KIT or PDGFRA, which plays an essential role in the development and progression of GIST (Heinrich *et al*, 2002, 2003). As GISTs are insensitive to conventional chemotherapy, the introduction of imatinib, a small-molecule receptor tyrosine kinase inhibitor active against KIT and PDGFRA, has been a major therapeutic breakthrough. Imatinib therapy has dramatically improved the survival of patients with unresectable or metastatic GIST (Verweij *et al*, 2004). Despite these successes, about 10% of the patients show initial resistance to imatinib. Moreover, complete remissions are almost never seen and most patients experience disease progression after a median period of approximately 2–3 years (Verweij *et al*, 2004). To date, sunitinib, an inhibitor of multiple receptor tyrosine kinases including KIT, PDGFRA, vascular endothelial growth factor receptor (VEGFR), and fms-related tyrosine kinase 3 (FLT3), is used as a second-line treatment providing clinical benefit in patients with imatinib-resistant GIST for a limited time period (Judson and Demetri, 2007). However, there is urgent need for the development of new therapeutics acting through pathways complementary to those targeted by KIT kinase inhibitors such as imatinib and sunitinib.

Fas (CD95) and Fas ligand (FasL; CD95L) belong to the TNF family of death receptors and ligands (Itoh *et al*, 1991; Suda *et al*, 1993). At the molecular level, binding of FasL to Fas induces receptor trimerisation, followed by the binding of Fas-associated death domain (FADD) with caspase 8 and/or 10 to the intracellular death domain of Fas. Caspase activation within this complex initiates cleavage and activation of an intracellular cascade of effector caspases (e.g., caspases 3, 6, and 7), eventuating in cleavage of specific death substrates and apoptosis (Timmer *et al*, 2002). Thus, in tumours expressing Fas, targeting of Fas-mediated apoptosis could be a promising therapy. Although many *in vitro* and *in vivo* cancer models have shown sensitivity towards Fas agonistic antibodies, clinical application of these antibodies is hampered because of severe liver toxicity (Ogasawara *et al*, 1993). Furthermore, resistance to Fas-mediated apoptosis because of inhibitory mechanisms along the apoptotic signalling pathway has been observed in other cancer models (Houston and O'Connell, 2004). Soluble forms of FasL (sFasL) are potentially less toxic (Schneider *et al*, 1998), and a hexameric form of sFasL, produced by fusing the dimer-forming serum protein stalk of human ACRP30 to the trimeric portion of human FasL, has recently been developed. This compound, called MegaFasL, is more cytotoxic to tumour cells compared to trimeric sFasL (Holler *et al*, 2003; Greaney *et al*, 2006; Etter *et al*, 2007). MegaFasL is currently tested in a phase-I clinical trial (ClinicalTrails.gov identifier: NCT00437736).

This study was initiated given the lack of data on expression of Fas and FasL in GIST. Therefore, the sensitivity of GIST cell lines towards Fas activation and the potentiating effect of imatinib on this activation were investigated. In addition, the expression of Fas and FasL in primary GIST samples was studied. Together, these data implicate Fas as a potential therapeutic target in GIST.

## Materials and methods

### Cell culture

The GIST cell line GIST882 was developed from a primary, untreated, GIST, with a homozygous *KIT* exon 13 mutation (Tuveson *et al*, 2001). Cells were maintained in RPMI 1640 (Invitrogen, Praisley, UK) supplemented with 15% heat inactivated fetal calf serum (FCS; Bodinco, Alkmaar, The Netherlands) and 1 mM L-glutamine (Invitrogen). The GIST cell lines GIST48 and GIST430 were established from tumours that were progressive during imatinib therapy after an initial clinical response (Bauer *et al*, 2006). GIST48 harbours a homozygous primary *KIT* exon 11 mutation and a heterozygous secondary exon 17 mutation. GIST430 has a heterozygous primary *KIT* exon 11 and a secondary heterozygous exon 13 mutation. The KIT-negative GIST430K- cell line was derived from GIST430 cells. The GIST48 and GIST430 cells were maintained in F-10 (Invitrogen) supplemented with 10 and 15% FCS, respectively, and 0.5% mito+ serum extender (VWR International, Roden, The Netherlands) and 1% bovine pituitary extract (VWR International). The cervical carcinoma cell line HeLa was maintained in 1 : 1 DMEM/HAM supplemented with 10% FCS.

### Flow cytometry

Fas membrane expression was determined in GIST cells by flow cytometry as described previously (De Groot *et al*, 2005). Phycoerythrin (PE)-conjugated mouse anti-Fas monoclonal antibody (clone DX-2, 1 : 10; BD Pharmingen, Alphen aan de Rijn, The Netherlands) was used. PE-conjugated mouse IgG_1_ (BD Pharmingen) served as an isotype control.

### Acridine orange apoptosis assay

Cells were treated with MegaFasL (kindly provided by Apoxis, Lausanne, Switzerland) for 6 h. To investigate the effect of MegaFasL on imatinib-induced apoptosis, cells were pretreated with imatinib (kindly provided by Novartis, Basel, Switzerland) for 24 h followed by 24 h incubation with MegaFasL without removing imatinib. Alternatively, cells were first treated with MegaFasL for 24 h with subsequent treatment with imatinib for 24 h. Control cells were incubated with DMSO, which was used as a solvent for imatinib, where appropriate, or a single drug. After these treatments, acridine orange (10 *μ*g ml^−1^) was added to each well to distinguish apoptotic cells from vital cells. Apoptosis was defined as the appearance of apoptotic bodies and/or chromatin condensation, using a fluorescence microscope. Results were expressed as the percentage of apoptotic cells in a culture by counting at least 300 cells per well. All apoptosis assays were performed three times in duplicate.

### Western blot analysis

After treatment with MegaFasL, as described above, GIST882 and GIST48 cells were lysed and the protein concentration was determined according to the Bradford method. Western blotting was performed as described previously (De Groot *et al*, 2005). Primary antibodies were goat anti-Fas (polyclonal, 1 : 400; Santa Cruz, Heerhugowaard, The Netherlands), mouse anti-FasL and mouse anti-caspase 3 (clone 33 and clone 19, respectively, 1 : 1000; BD Transduction Laboratories, Alphen aan den Rijn, The Netherlands), rabbit anti-cleaved caspase 3 and rabbit anti-caspase 6 (1 : 1000; Cell Signalling Technology, Bioké, Leiden, The Netherlands), mouse anti-caspase 8 (clone 1C12, 1 : 500; Cell Signalling), and mouse anti-actin (clone C4, 1 : 20 000; ICN Biomedicals, Zoetermeer, The Netherlands).

### Tissue collection

The study group consisted of 45 primary GISTs derived from 44 patients. Paraffin-embedded tumour tissues of primary GISTs were retrieved from the files of the Department of Pathology of the University Medical Center Groningen. Tumours were surgically resected between 1985 and 2005. All tumours were reviewed by a pathologist with special expertise in soft tissue tumours (AJHS). The diagnosis of GIST was based on the typical histological features of a cellular spindle cell or epithelioid mesenchymal tumour of the gastrointestinal tract. In addition, all tumours were positive for CD117 (KIT) by immunohistochemistry. Tumours were categorised into risk groups, based on tumour size and mitotic index, according to the consensus classification for prediction of metastatic potential (Fletcher *et al*, 2002). Tumour size was obtained from the pathology reports. Mitotic index was counted in 50 consecutive high power fields ( × 40 objective; field diameter 0.55 mm). Clinicopathological data collected included sex and age of the patient at diagnosis, primary tumour site, and histological subtype. For the detection of *KIT* mutations, genomic DNA was extracted from paraffin-embedded tumour samples and *KIT* exon 9 or 11 was amplified by PCR. Both forward and reverse PCR products were sequenced and the results were compared with normal sequences. One patient had two primary GISTs, which were a high risk epithelioid gastric tumour lacking a *KIT* exon 9 and 11 mutation and a low risk spindle-cell small intestine tumour with a *KIT* exon 11 mutation, respectively. Patient and tumour characteristics are summarised in Table 1.

For the detection of Fas and FasL by western blotting, six frozen primary GIST samples were pulverised and dissolved in ice-cold PBS. After centrifugation at 18 000 **g** for 1 min to remove debris, the supernatant was collected. Western blot analysis was performed as described above.

All tumour samples used in this study were handled according to the guidelines of the Dutch Federation of Biomedical Scientific Societies (FMWV) as described in ‘Code Proper Secondary Use of Human Tissue’.

### Tissue microarray construction

Representative regions of the paraffin-embedded primary tumours were selected using H&E-stained slides and arrayed into a tissue microarray (TMA), as described previously (Westra *et al*, 2005). Briefly, three 0.6 mm tissue cores were taken from (distinct) representative areas of each tumour specimen using a manual tissue arrayer (Beecher Instruments, Sun Prairie, WI, USA) and then transferred to a standard-size recipient paraffin block. The array contained 161 tissue cores including 45 tumour samples in triplicate and 13 normal tissues in duplicate, the latter serving as an internal control for immunohistochemistry.

### Immunohistochemistry

Sections of 4 *μ*m were taken from each array block and deparaffinised in xylene. Immunohistochemistry was performed as previously described (Arts *et al*, 2005). Antigen retrieval was carried out by autoclaving sections in blocking solution (2% blocking reagent (Roche Diagnostics, Mannheim, Germany) and 0.2% SDS in maleic acid buffer (pH 6.0)) at 115°C for three times 5 min. Primary antibodies were mouse anti-Fas (clone CH-11, 1 : 100; Upstate Biotechnology, Lake Placid, NY, USA) and mouse anti-FasL (clone 33, 1 : 160; BD Transduction Laboratories). As a negative control, a serial section was processed without the addition of primary antibody. Normal tissue samples within the TMA block derived from liver and kidney served as positive controls for Fas and FasL staining (Leithauser *et al*, 1993; Lee *et al*, 1999).

### Staining analysis

Immunohistochemistry results were scored independently by two observers (BR and AJHS) without knowledge of the clinicopathological data. As in each individual core all tumour cells showed the same staining, cores were scored in a semiquantitative manner for staining intensity: no staining (0), weakly positive staining (1), positive staining (2), strong positive staining (3). Discrepant scoring results were discussed under a multiheaded microscope to achieve consensus. If cores from one tumour differed in staining intensity, the median score of the three related cores determined the score of the tumour. Furthermore, the staining pattern was determined from each core.

### Statistical analysis

Data analysis was performed using SPSS 12.0 software package for windows (SPSS Inc., Chicago, IL, USA). Differences in distributions of Fas or FasL staining intensity and the different tumour characteristics were evaluated by the *χ*^2^ test. To determine the correlation between Fas and FasL staining, the spearman's rank test was used. A two-tailed *P*-value <0.05 was considered to be significant.

## Results

### MegaFasL-induced apoptosis in GIST cells

**Figure 1:**
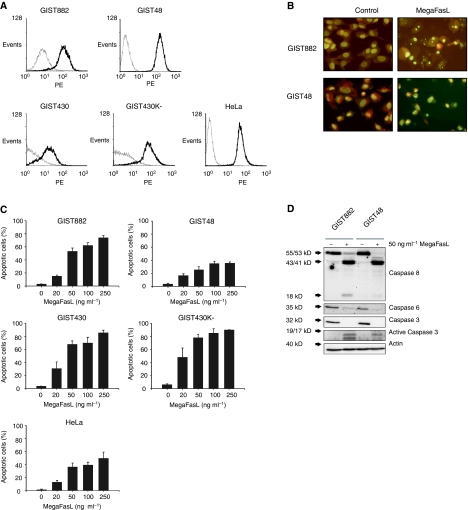
Apoptosis induction by MegaFasL in GIST cell lines. (**A**) Analysis of Fas membrane expression by flow cytometry. The thin grey line represents the IgG control and thick black line reflects the anti-Fas antibody. HeLa was used as a positive control. (**B**) Representative images of acridine orange staining of GIST882 and GIST48 with or without treatment with 50 ng ml^−1^ MegaFasL for 6 h. Nuclear fragmentation is seen in cells treated with MegaFasL. (**C**) GIST cell lines, and HeLa, were incubated with different concentrations of MegaFasL for 6 h. Apoptosis was quantified with acridine orange staining. Data represent the mean±s.d. of three independent experiments. (**D**) Expression of caspases 8, 6, 3, and cleaved caspase 3 after treatment of GIST882 and GIST48 cells with 50 ng ml^−1^ MegaFasL for 6 h, analysed by western blot. Immunoblotting of actin was used as a loading control.

To evaluate whether Fas could be used as a target in GIST, we first investigated Fas membrane expression in a panel of imatinib-sensitive (GIST882) and imatinib-resistant (GIST48, GIST430 and GIST430K-) cell lines. The cervical carcinoma cell line HeLa is responsive to MegaFasL and was used as positive control (Holler *et al*, 2003). Flow cytometry analysis revealed that all the GIST cell lines had a high level of Fas membrane expression (Figure 1A). We therefore tested the effectiveness of MegaFasL, a hexameric form of sFasL. After as little as 6 h of MegaFasL treatment, dose-dependent apoptosis was observed in all the GIST cell lines. MegaFasL induced substantially higher levels of apoptosis in GIST882, GIST430, and GIST430K- than in HeLa, while nearly equal amounts were observed in GIST48 compared to HeLa (Figure 1B and C). Treatment of GIST882 and GIST48 with MegaFasL resulted in caspase 8 activation and the disappearance of the inactive proform of caspases 3 and 6. The active p19/p17 fragments of caspase 3 were detected in both cell lines, although to a lesser extent in GIST48, which is in agreement with the observed difference in apoptosis levels between these two cell lines when treated with 50 ng ml^−1^ MegaFasL (53% apoptosis in GIST882 and 25% in GIST48; Figure 1C and D). The effect of MegaFasL was dependent on caspase activation as zVAD-fmk, a pan-caspase inhibitor, completely blocked apoptosis induction by this compound (data not shown).

### The effect of MegaFasL on imatinib-induced apoptosis

**Figure 2:**
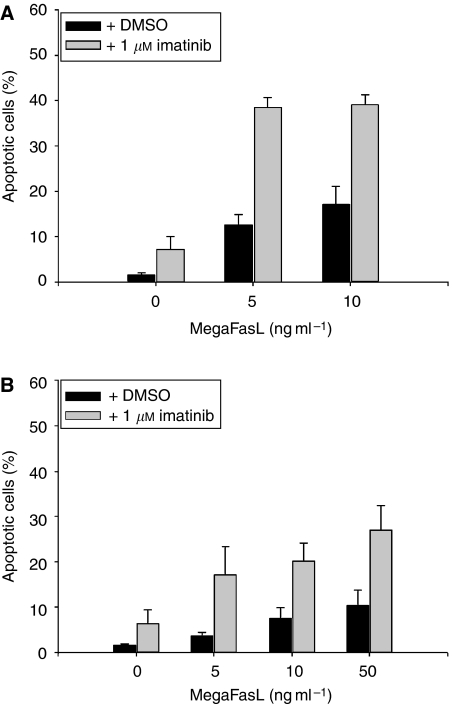
The effect of MegaFasL on imatinib-induced apoptosis. (**A**) GIST882 cells were pretreated with MegaFasL, as indicated, for 24 h followed by incubation with either DMSO-only or 1 *μ*M imatinib for an additional 24 h. Apoptosis was determined by acridine orange staining. (**B**) GIST48 cells were treated with MegaFasL and/or imatinib, as described in (**A**). Data represent the mean±s.d. of three independent experiments.

Following the identification of MegaFasL as a potent apoptosis-inducing agent in GIST cells, we investigated its effect in combination with imatinib. GIST882 pretreatment with MegaFasL followed by the addition of imatinib, appeared to be the most effective schedule. In this way, low concentrations of MegaFasL for 24 h followed by the addition of imatinib for another 24 h substantially increased the amount of apoptosis compared to levels seen with either MegaFasL or imatinib treatments alone (Figure 2A). When imatinib was administered before MegaFasL, no synergistic but rather an additive effect was observed (data not shown).

Recently, Bauer *et al* (2006) showed that GIST48 cells are relatively resistant towards imatinib. We therefore also evaluated the combination of MegaFasL and imatinib in GIST48 by using the same treatment schedule as GIST882. As in GIST882, synergistic apoptosis induction was seen for the combination of MegaFasL and imatinib, although higher concentrations of MegaFasL were necessary to induce a substantial amount of apoptosis (Figure 2B).

### Fas and FasL expression in GIST by immunohistochemistry

As MegaFasL appeared to be an active agent in GIST cells, we studied the expression of Fas and FasL in 45 GIST samples by immunohistochemistry using a TMA. Table 2 shows the overall staining characteristics of Fas and FasL in the 45 GISTs tested. Fas was detectable in all the tumour samples studied and was strongly expressed in 62%. FasL expression was discerned in 89% of the tumours with 27% staining strongly positive. A significant correlation between Fas and FasL expression was found (spearman's correlation coefficient=0.4, *P*=0.006). Further statistical analysis revealed that Fas and FasL staining intensity was not associated with (1) risk classification, (2) primary site, (3) histological subtype, (4) tumour size, (5) mitotic index, and (6) *KIT* mutation status.

**Figure 3:**
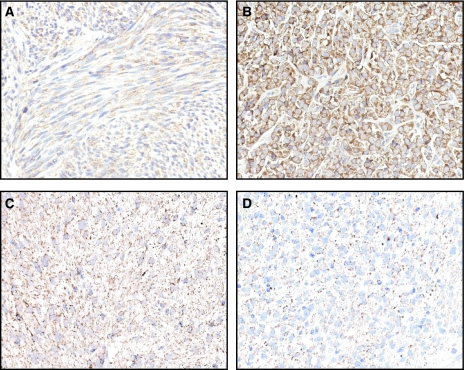
Immunohistochemical staining for Fas and FasL in paraffin-embedded GIST samples. Representative examples of immunostaining for Fas (**A** and **B**) showing predominantly diffuse cytoplasmic staining and FasL (**C** and **D**) showing granular cytoplasmic staining (magnification, × 400).

Both Fas and FasL immunohistochemical staining was predominantly cytoplasmic. The staining pattern for Fas was diffuse, in contrast to FasL, which was mainly granular (Figure 3).

### Fas and FasL expression in GIST by western blot analysis

**Figure 4:**
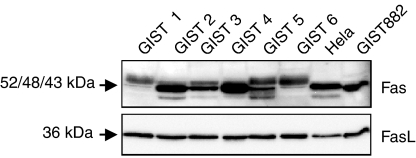
Expression of Fas and FasL in six resected GISTs and the GIST882 cell line as determined by western blot analysis. HeLa served as a positive control. GIST 2, 3, and 4 harbour a *KIT* exon 11 mutation, GIST 1, 5, and 6 have no *KIT* exon 9 or 11 mutations.

In addition to immunohistochemistry, Fas and FasL protein expression in six GIST samples and the GIST882 cell line was evaluated by western blot analysis. HeLa was used as a positive control (Hougardy *et al*, 2005). As shown in Figure 4, protein products corresponding to the predicted molecular mass of ∼36 kDa for FasL were detected in HeLa, GIST882, and in all six GISTs. The ∼48 kDa Fas protein product was also detectable in GIST882 and in all GIST samples. Furthermore, most GIST samples – as well as the HeLa cell line – showed a lower band of ∼43 kDa. In addition, most of the GISTs featured an additional band that migrated more slowly than the two bands shown in HeLa (Figure 4). This additional ∼52 kDa band could represent a highly sialylated but functional form of Fas (Oehm *et al*, 1992; Keppler *et al*, 1999).

## Discussion

The results of this study indicate that Fas may be a promising therapeutic target in GIST. The GIST cell lines in our panel were found to be highly sensitive to MegaFasL, which proved to be an active apoptosis-inducing agent. Moreover, MegaFasL sensitised GIST cells to imatinib-induced apoptosis. In addition to these *in vitro* data, we found that each of 45 primary GISTs expressed Fas, and most expressed FasL.

This is the first study investigating the role of death receptors in the treatment of GIST. Both imatinib-sensitive (GIST882) and imatinib-resistant GIST cell lines (GIST48, GIST430, and GIST430K-) were responsive to MegaFasL. Furthermore, MegaFasL sensitivity appeared to be independent of KIT expression because the GIST430 cell line that expresses KIT was as sensitive to MegaFasL as its KIT-negative daughter cell line. We found that low concentrations of MegaFasL sensitised GIST cells to imatinib-induced apoptosis. Notably, it appeared that the sequence of drug delivery was important. Pretreatment of cells with imatinib followed by incubation with MegaFasL had no more than an additive effect. Reversing this schedule, however, resulted in synergistic apoptosis induction. The underlying mechanism for this observation remains unclear. Nimmanapalli *et al* (2001) showed that the sensitising effect of imatinib to TRAIL in Bcr-Abl-positive leukaemia cells was reduced when TRAIL was administered after exposure to imatinib. Although imatinib causes cell cycle arrest, it is unlikely that this mechanism is involved in the reduced potentiation of MegaFasL-induced apoptosis by imatinib pretreatment, as Fas-mediated apoptosis is not cell cycle dependent (Hueber *et al*, 1998; Tepper *et al*, 2000). Possibly, KIT inhibition by imatinib alters the membrane distribution of Fas, making it less accessible for MegaFasL. Alternatively, the activated caspase cascade by MegaFasL may induce cleavage of proteins involved in cellular resistance to imatinib.

In our patient cohort, which was representative for an unselected GIST population with regard to sex, age, tumour localisation, histology, risk classification, and *KIT* exon 11 mutations (Corless *et al*, 2004; Nilsson *et al*, 2005), Fas and FasL were abundantly expressed. Western blot analysis confirmed these findings. Acquisition of FasL expression by tumour cells is one of the mechanisms involved in tumour immune escape. Various tumour types have been reported to express FasL, which can be negatively correlated with prognosis as described for colon and breast carcinomas (Reimer *et al*, 2000; Belluco *et al*, 2002). In our study, 89% of the GISTs expressed FasL, but no correlation was found with tumour size and mitotic index or risk groups based on these prognostic factors, which predict metastatic behaviour. The FasL staining pattern in the cytoplasm was granular, an expression pattern that has also been observed in ovarian carcinoma and melanoma, where FasL is stored in cytoplasmic microvesicles and, upon release, induces apoptosis in Fas-bearing immune cells (Andreola *et al*, 2002; Abrahams *et al*, 2003; Arts *et al*, 2005). FasL could therefore be involved in the protection of GIST cells against the immune system. In addition, we found a significant correlation between Fas and FasL expression in GIST, which has been observed in many other tumour types as well (Arts *et al*, 2005). This co-expression has been implicated in tumour progression, as FasL can act in an autocrine or juxtacrine Fas-dependent manner to promote tumour growth (Lambert *et al*, 2003; Houston and O'Connell, 2004). Further studies are needed to determine whether such mechanisms also apply in GIST.

Gastrointestinal stromal tunour patients generally respond to therapy with imatinib, which inhibits the KIT and PDGFRA oncoproteins that appear to be initiating oncogenic events in most GISTs. However, in the long run, most patients develop resistance to imatinib therapy, as manifested by tumour progression. It appears that, although imatinib treatment induces apoptosis and causes cell cycle arrest of GIST cells, a fraction of the cells usually survive, and these surviving cells may subsequently form the nidus of an imatinib-resistant GIST, often containing secondary KIT mutations (Antonescu *et al*, 2005; Heinrich *et al*, 2006). Therefore, novel systemic therapeutic approaches are needed to maximise GIST cell death. Targeting death receptors, such as Fas, is a promising anticancer strategy by which apoptotic cell death can be induced. Unfortunately, the introduction of therapies targeting Fas with agonistic antibodies has been hampered by liver toxicity (Ogasawara *et al*, 1993). However, the recently developed MegaFasL is potentially less toxic and has been shown to be active in several *in vitro* and *in vivo* models (Holler *et al*, 2003; Greaney *et al*, 2006; Etter *et al*, 2007). MegaFasL is formed by crosslinking two sFasL trimers resulting in a hexameric protein that much more efficiently induces clustering of Fas on the cell surface, leading to a higher degree of multimerisation of activated receptors as compared with sFasL. As a consequence, a high local concentration of the intracellular death domains might be formed, leading to more efficient activation of caspase 8 after binding to the adaptor molecule FADD (Holler *et al*, 2003).

We found that very low doses of MegaFasL potentiate the effect of imatinib. Therefore, even when the therapeutic window is low, systemic use of MegaFasL could still have a beneficial effect in combination with imatinib. As many GISTs tend to metastasise in the abdominal cavity, potential liver toxicity by MegaFasL might be circumvented by intraperitoneal applications to prevent MegaFasL from reaching high levels in the circulation (Stewart *et al*, 2002; Etter *et al*, 2007). In this way, MegaFasL might be applied in combination with systemic imatinib. The abundant Fas expression in GISTs suggests that also MegaFasL alone might be an option for patients with primary or acquired resistance to imatinib. However, besides Fas membrane expression, one should take in account the expression of intracellular components affecting sensitivity to Fas-mediated apoptosis in these GISTs.

In conclusion, we have identified Fas as a uniformly expressed receptor in GIST, which may be used as a therapeutic target. This is supported by our *in vitro* studies, in which MegaFasL is a potent apoptosis-inducing agent that is even more efficient in combination with imatinib.

## Figures and Tables

**Table 1:** Patient (*n*=44) and tumour (*n*=45) characteristics

	***n* (%)**
*Sex*	
Male	19 (43)
Female	25 (57)
Age (years), median (range)	65.5 (36–87)
	
*Risk classification*	
Very low risk	4 (9)
Low risk	13 (29)
Intermediate risk	13 (29)
High risk	15 (33)
	
*Primary site*	
Stomach	29 (64)
Small intestine	12 (27)
Colon	3 (7)
Unknown	1 (2)
	
*Histology*	
Spindle cell	37 (82)
Epithelioid	7 (16)
Mixed	1 (2)
	
*Tumour size*	
⩽5 cm	19 (42)
5–10 cm	17 (38)
>10 cm	9 (20)
	
*Mitotic figures*	
⩽5 per 50 HPF	38 (84)
>5 per 50 HPF	7 (16)
	
*KIT mutation*	
Exon 11 mutation	28 (62)
Exon 9 mutation	1 (2)
No exon 11 or 9 mutation	15 (33)
Unknown	1 (2)

Abbreviation: HPF=high power fields.

**Table 2:** Expression of Fas and FasL in GIST

**Staining intensity**	**Fas, *n* (%)**	**FasL, *n* (%)**
0	0 (0)	5 (11)
1	3 (7)	16 (36)
2	14 (31)	12 (27)
3	28 (62)	12 (27)
		
Total	45 (100)	45 (100)
